# Highly angle-sensitive and efficient optical metasurfaces with broken mirror symmetry

**DOI:** 10.1515/nanoph-2022-0793

**Published:** 2023-02-13

**Authors:** Nayoung Kim, Myungjoon Kim, Joonkyo Jung, Taeyong Chang, Suwan Jeon, Jonghwa Shin

**Affiliations:** Department of Materials Science and Engineering, Korea Advanced Institute of Science and Technology, Daejeon 34141, Republic of Korea

**Keywords:** angle-multiplexed, beam deflection, broken symmetry, effective medium, inverse design, metalens

## Abstract

Optical metasurfaces have great potential to overcome the functional limitations of conventional optical devices. In addition to polarization- or wavelength-multiplexed metasurfaces, angle-multiplexed metasurfaces can provide new degrees of freedom, enabling previously unrealized complex functionality in diverse applications such as LiDAR, augmented reality glasses, and imaging. However, there have been fundamental trade-offs in transmission efficiency and angular sensitivity for practically important paraxial rays. In this paper, we overcome this limitation by breaking mirror symmetries of single-layer metasurface structures. Based on an effective medium theory, we intuitively explain which material parameters affect the sensitivity and efficiency and prove that high sensitivity and high efficiency can be achieved simultaneously by breaking the mirror symmetry. Based on this, we propose optimized metasurfaces for two applications: an angle-multiplexed beam-steering device with up to 93% relative efficiency and an angle-multiplexed metalens array that can break the fundamental resolution–density trade-off of microlens arrays with high efficiency. The proposed angle-selective designs could pave the way for the development of new classes of compact optical devices with novel functions.

## 1 Introduction

Recent advances in optical nanostructures have enabled pixel-wise, precise phase control of transmitted light with functionalities not found in conventional optical materials and devices. Multiplexed metasurfaces are representative examples of such advancements, and several recent works have demonstrated metasurfaces that purposefully exhibit different behaviors depending on the properties of the incident light. Many proposals have focused on polarization- or wavelength-multiplexed responses such as polarization-sensitive holograms [1–4], beam splitters [2, 5], polarimeters [6–9], and multi-wavelength metasurfaces for color routings [10–12], and wavelength-multiplexed holograms [13, 14]. More recently, angle-multiplexed metasurfaces are emerging because of their unique potentials. Conventionally, diffraction gratings and total internal reflection in prisms have been widely used for angle-selective beam control or precise measurement of angular properties. However, angle-multiplexed metasurfaces can possess even more degrees of freedom for transmission phase engineering and have been realized with several different physical principles, such as localized resonance [15], high-order diffraction [16], guided wave excitations [17], near-field coupling between neighboring nanostructures [18], and surface plasmon polaritons [19]. Despite these pioneering studies, two difficult hurdles should be overcome before wide applications of the concept: namely, low angular sensitivity (required incident angle difference is larger than ∼10° in many cases) [15, 16, 18, 19] or low optical efficiency (as low as 10% even in recent papers) [17]. It appears even more challenging to achieve high efficiency and high sensitivity at the same time, especially for optical beams with near-zero incidence angles. This compromises many practical applications that utilize paraxial rays around the zero incident angle.

In this work, we propose single-layer metasurfaces with broken mirror symmetry as a potential route to solving all the above problems. First, we investigate a class of periodic metasurfaces with slanted motifs as anisotropic effective media and theoretically explicate why the broken symmetry is essential for attaining simultaneously high angular sensitivity and efficiency at near-zero incidence angles. Then, we construct gradient metasurfaces with new functionalities using an adjoint method for the optimal arrangement of the proposed motifs and demonstrate their new functionalities. Two such gradient metasurface examples are presented: (1) an angle-multiplexed beam-steering device with up to 93% relative efficiency and (2) an angle-multiplexed metalens array that can break the fundamental resolution–density trade-off of current microlens arrays with high efficiency. Angular multiplexing provides new degrees of freedom in optical devices, making previously unavailable functionality possible.

## 2 Angle-sensitive anisotropic media

**Figure 1:**
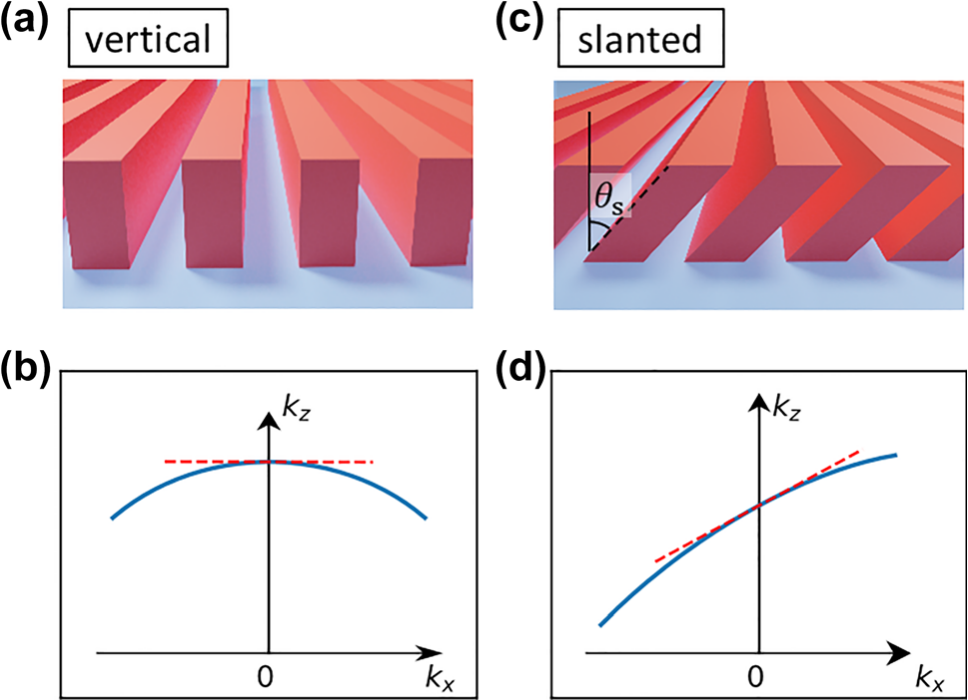
Concept of symmetry and broken mirror symmetry structure. (a) and (c) Schematic of periodic structures extended infinitely along the *y*-axis: (a) vertical and (c) slanted grating. The slanted grating structure has broken mirror symmetry plane normal to the *z*-axis. (b) and (d) The equi-frequency contours of the structures in a periodic array of subwavelength motifs. The graphs correspond to (b) vertical and (d) slanted structures. The dashed line illustrates the phase gradient at zero incidence angle. Because the slanted structure has a slope in the graph, it has nonzero phase gradient around zero incidence angle and has higher angle-sensitivity for paraxial wave incidences.

Due to their simple design and fabrication, most previous optical metasurfaces utilized vertical posts or grating structures that have a mirror symmetry plane normal to the vertical axis (denoted as the *z*-axis hereafter). In reciprocal systems (e.g., linear, non–magneto-optic, and time-invariant systems), this dictates that two input beams whose incident angles are opposite to each other have exactly the same transmission coefficient in both their magnitudes and phases [20]. Due to this symmetric scattering response, the gradient of the transmission phase as a function of the in-plane wavevector has to be zero when the incidence angle is zero (i.e., normal incidence), as illustrated in Figure 1(a) and (b), which limits the angular sensitivity of such metasurfaces at near-zero incidence angles.

On the other hand, multilayer structures with no such mirror symmetry can exhibit asymmetric transmission for the opposite incidence angles (*θ* and *−θ*) owing to their off-diagonal electric susceptibilities [17, 20]. Moreover, they can have a nonzero phase gradient around zero incidence angle (Figure 1(c) and (d)), which is crucially important for practical angle-sensitive devices operating on paraxial rays. If a single-layer metasurface can overcome the symmetry-imposed constraints explained above, this would have an even higher potential impact since it does not require alignment accuracy at the nanometer scale and multiple lithographic masks. We show that a single-layer metasurface with slanted walls can break mirror symmetry and possess a large gradient of the transmission phase even at a normal incidence angle. We show that a homogeneous, anisotropic effective medium model can explain the optical properties of such metasurfaces accurately and compare its performance to that of vertical structures in terms of their angular sensitivity.

### 2.1 Comparison of vertical and slanted structures

**Figure 2:**
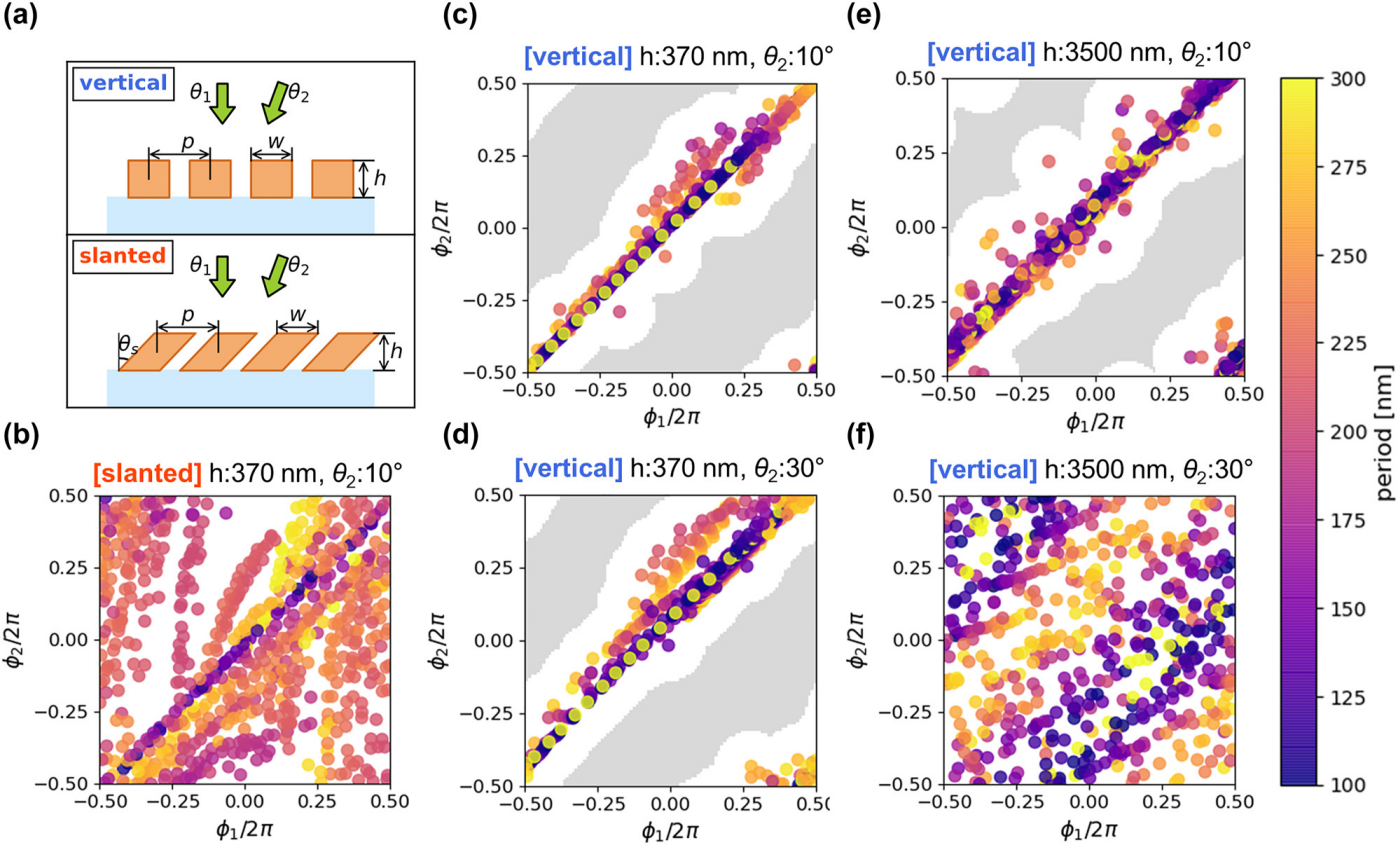
Comparison of vertical and slanted structure in the view point of transmission phase combinations. (a) Schematic of structures for simulation. The TiO_2_ gratings on the glass substrate are periodic in *x*-direction and extended and infinitely along the *y*-direction. Input waves are *p*-polarized and in the air. All the transmission combinations are calculated with varying the period (*p*) and width (*w*) of the gratings with fixed other geometrical parameters. (b) Combinations of transmission phases for the slanted grating structure. (c)–(f) Combinations of transmission phases for the vertical grating structures. The color represents the period of the structures with different width parameters. The gray-shaded region indicates that no database can realize the intended pair of phase values within a *π*/4 rad error. The specified values within each subpanel of the figure.

We consider slanted structures with a parallelogram-like cross section that can be fabricated by angled deposition [21, 22] or angled reactive ion-beam etching [23, 24]. In the following analysis, we enforce uniformity in the *y*-direction (one of the in-plane directions) to induce only the minimal set of symmetry breaks required to enhance the angular sensitivity and simplify the analysis and discussion. But the conclusion could be generalized to structures for which the continuous translational symmetry in the *y*-direction is also broken. The structural parameters investigated are shown in Figure 2(a).

For incident angle-sensitive beam steerers, metalenses, or other types of metasurfaces that can manipulate two incident beams at different incident angles in very different and independent ways, we need unit structures with diverse combinations of transmission phases (*ϕ*_1_, *ϕ*_2_) for the two input beams. For example, to achieve 6-level (e.g., 0, *π*/3, 2*π*/3, *π*, 4*π*/3, 5*π*/3) phase control for each input beam independently, at least 36 candidate structures with (*ϕ*_1_, *ϕ*_2_) values representing all possible combinations of such phase values are required. For devices with low noise and high efficiency, it may be desirable to have finer level of phase control, in which case even more diverse phase combinations are needed, preferably spread across the entire phase space in an even spacing. In practice, it is also important that such a set of candidate structures have the same height (*h*) and slant angle (*θ*_s_), as it will allow fabrication of such devices with a single lithographic mask, eliminating the need for nanoscale mask alignments.

Figure 2 demonstrates that slanted structures make it much easier to ensemble such a set of structures for near-zero incident angles. We assumed that both the slanted and vertical structures are made of an optically isotropic material with a refractive index of 2.7 that has been deposited on a glass substrate. Individual fin structures with a common width *w* and thickness *h* are periodically placed with a period *p* along the *x*-direction. For slanted structures, the slant angle *θ*_s_ provides an additional degree of freedom. Figures 2(b)–(f) show the resulting transmission phases (*ϕ*_1_, *ϕ*_2_) for 400 nm wavelength light for 693 different combinations of *p* and *w* in each subpanel. Here, *p* and *w* are varied within the range of 100–300 nm and 0.1*p*–0.9*p*, respectively, while *h* and *θ*_s_ are kept constant at the specified values within each subpanel of the figure. To illustrate the angular sensitivity near-zero incident angle, one of the incident angles (*θ*_1_) is set to be zero and the other angle (*θ*_2_) is chosen to be 10°, with the electric field is within the *x*–*z* plane (*p*-polarized). For vertical structures, *θ*_2_ = 30° cases are also included in the comparison because of their lower angular sensitivity.

The significant difference in angular sensitivity between vertical and slanted structures is apparent in the degree of diversity in the transmission phase combinations. For the same thickness *h* = 370 nm, the achievable combinations of (*ϕ*_1_, *ϕ*_2_) for slanted structures (*θ*_s_ = 37°) are spread across the phase space, while those for vertical structures are mostly concentrated along the diagonal line of *ϕ*_1_ = *ϕ*_2_. This indicates that the transmission phases through the slanted structures can be tuned to very different values for two incident beams separated by only 10° in their incident angles, indicating high angular sensitivity. However, the vertical structures cannot impart significantly different phases for those two beams in most cases.

Quantitatively, a Delaunay triangulation of the above points can be performed to determine the radii of the circumcircles, which shows how large the voids are in the phase space [Supporting Information]. The radii of the circumcircles also relate to the potential phase errors of a metalens made of these structures, which are defined as the distance of a target phase combination in the two-dimensional phase space to the nearest point realized by a known (*p*, *w*) pair. For the slanted structures with a 37° slanted angle (*θ*_s_), the maximum radius of such circumcircles (hence, the maximum phase error) is 0.215*π* rad (38.7°). This maximum radius is almost three times smaller than 0.622*π* rad (112°) for vertical structures whose metasurface layers have the same thickness (370 nm) and incident angles (0° and 10°). In fact, this specific value of 37° slanted angle is obtained through an iterative optimization process trying to minimize the maximum phase error and will be different for other choices of *h* or *θ*_2_. Moreover, for more than 49% of the 2D phase space area (the gray-shaded region in Figure 2(c)), the target phase combination does not have a corresponding vertical structure design within the database that can realize the intended phase value pairs within *π*/4 rad (45°) error. This implies that slanted structures could serve as versatile building blocks for efficient, angle-sensitive metasurfaces, while vertical structures may not be as ideal because of many unwanted scattering can occur due to large phase errors.

For the vertical structures, one can try diversifying the achievable (*ϕ*_1_, *ϕ*_2_) combinations by using an incident angle separation three times as large (*θ*_2_ = 30°, Figure 2(d)) or a metasurface-layer thickness more than nine times as large (*h* = 3500 nm, Figure 2(e)) than the case in Figure 2(c). However, the resulting data reveal that these constraint relaxations are insufficient individually: when the two relaxations are combined (*θ*_2_ = 30°, *h* = 3500 nm, Figure 2(f)), most of the phase space can be covered with vertical structures. Therefore, it is apparent that the slanted structures open the possibilities for high-sensitivity and high-efficiency angle-multiplexed metasurfaces with much more compact designs for paraxial rays. We note that the results can be generalized to other incidence angles, with different optimal slanted angles than in the example above, but the design principle remains the same.

### 2.2 Effective medium approximation

The significant difference in angle sensitivity for paraxial rays between the vertical and slanted structures, as phenomenologically illustrated above, results from the broken mirror symmetry of the unit structures, and it is intuitively understood when considering the optical properties of anisotropic media. A periodic array of subwavelength motifs can be approximated as a uniform effective medium, which can be used to model both vertical and slanted structures. The difference is that vertical gratings or vertical posts map to anisotropic effective materials with optical axes normal to the substrate [*θ*_c_ = 0 in Eq. (1)], while slanted ones have an effective optical axis that is slanted. In either case, the permittivity tensor of anisotropic media can be expressed in Cartesian coordinates aligned with the substrate by the product of rotation matrices ( $\bar{\bar{R}}\left(\theta_{\text{c}}\right)$ and ${\bar{\bar{R}}}^{T}\left(\theta_{\text{c}}\right)$ ) and a diagonal matrix ( $\bar{\bar{\varepsilon }}$ ) whose diagonal elements are the permittivity values (*ɛ*_1_, *ɛ*_2_, and *ɛ*_3_) along the three orthogonal axes:

(1) $$\begin{array}{c} {\bar{\bar{\varepsilon }}}_{R}=\bar{\bar{R}}\left(\theta_{\text{c}}\right)\cdot \bar{\bar{\varepsilon }}\cdot {\bar{\bar{R}}}^{T}\left(\theta_{\text{c}}\right)=\left(\begin{array}{ccc} \varepsilon_{xx} & \varepsilon_{xy} & \varepsilon_{xz}\\ \varepsilon_{yx} & \varepsilon_{yy} & \varepsilon_{yz}\\ \varepsilon_{zx} & \varepsilon_{zy} & \varepsilon_{zz} \end{array}\right). \end{array}$$

$\bar{\bar{R}}\left(\theta_{\text{c}}\right)$ rotates the crystal axis by an angle *θ*_c_ with respect to the *y*-axis as we choose our coordinates in such a way that the slanting occurs on a plane perpendicular to the *y*-axis (Figure 1). The permittivity tensor satisfies the reciprocity condition ${{\bar{\bar{\varepsilon }}}_{R}}^{\text{T}}={\bar{\bar{\varepsilon }}}_{R}$ if we assume reciprocal media, which is a valid approximation for common dielectric, semiconducting, or metallic materials without a strong magneto-optic effect or nonlinearity. We investigate *p*-polarized wave propagation through slanted gratings that extend infinitely along the *y*-axis; therefore, only permittivity indices *ɛ*_*xx*_, *ɛ*_*zz*_, and *ɛ*_*xz*_ need to be considered. The diagonal components are defined as *ɛ*_1_ and *ɛ*_3_, and the nonmagnetic material is assumed to have permeability *μ*_*y*_ = 1.

**Figure 3:**
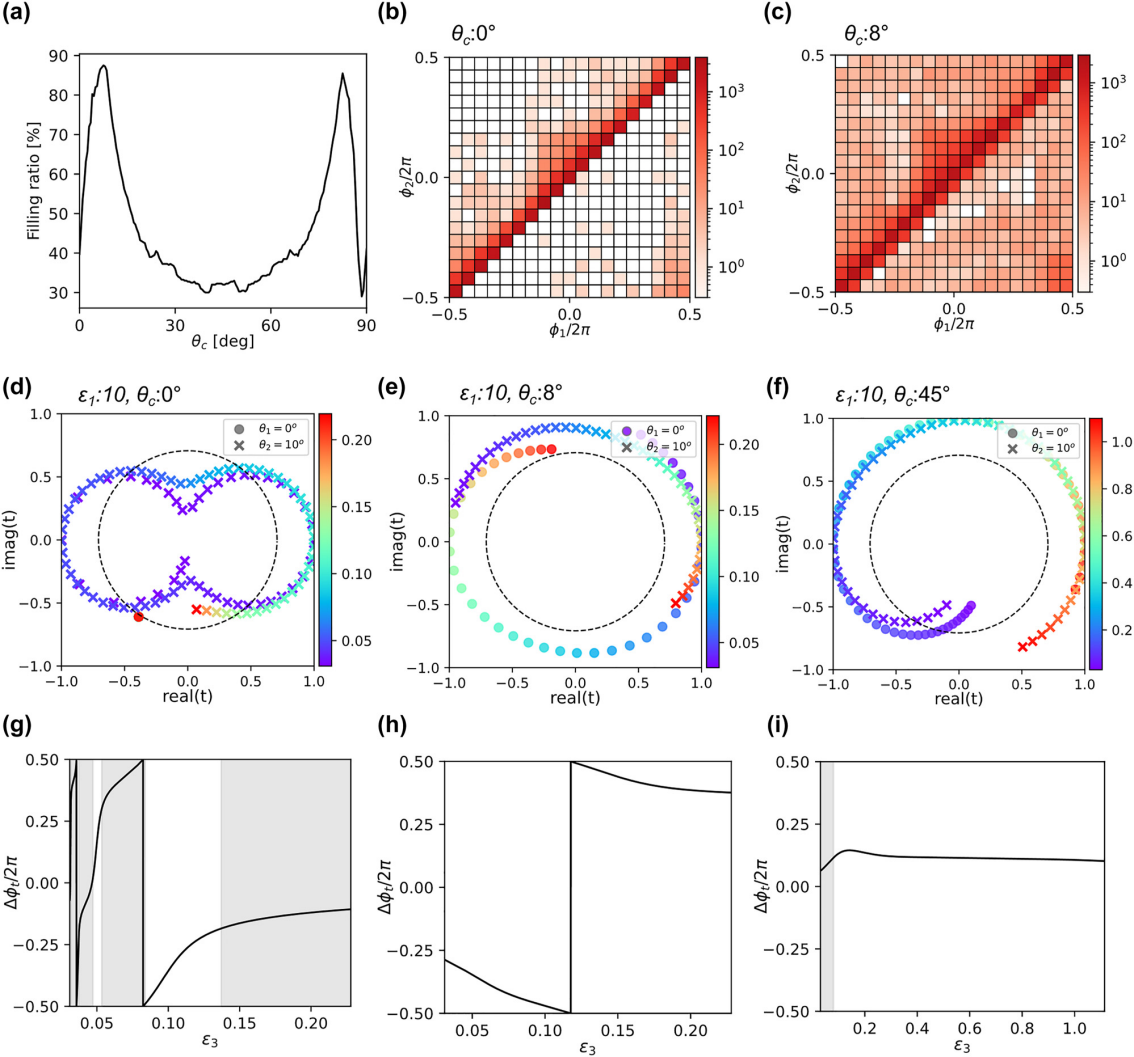
Theoretical analysis based on effective medium approach. (a)–(c) Possible transmission phase combinations for the uniform anisotropic medium with various values of permittivities (ϵ_1_ and ϵ_3_). (a) The filling ratio of the phase map with the 10^5^ number of permittivity combinations for a given *θ*_c_. (b) and (c) The density map of the two-dimensional phase space (for fixed two inputs: *θ*_1_ = 0° and *θ*_2_ = 10°) for two representative values of (b) *θ*_c_ = 0° and (c) *θ*_c_ = 8°. (d)–(f) The transmission coefficients of the anisotropic medium with different slanted optical axis (d) *θ*_c_ = 0° (e) *θ*_c_ = 8° (f) *θ*_c_ = 45° in complex domain as a function of ϵ_3_. The ϵ_1_ is fixed to 10. The values for the different inputs are denoted with different markers. The dashed circles denote the threshold of transmission amplitude (|*t*|^2^ = 0.5). (g)–(i) The corresponding phase difference as a line graph for (g) *θ*_c_ = 0°, (h) *θ*_c_ = 8°, (i) *θ*_c_ = 45°. The shaded regions indicate where the transmittance is lower than 0.5 at least for one of the incident angles.

Figure 3(a)–(c) depict possible transmission phase combinations for a slab made with such an anisotropic medium for various values of permittivities (*ɛ*_1_ and *ɛ*_3_), in a similar fashion to Figure 2. The transmission amplitude and phase are analytically calculated using a transfer-matrix method [25, 26], and the details are given in the Supporting Information. To investigate how diverse combinations of such a slab can achieve two different near-zero incidence angles, we fix the wavelength of the incident light (400 nm), the angles of incidence (*θ*_1_ = 0*°*, *θ*_2_ = 10*°*), and the slab thickness (300 nm) and varied the permittivity values from 0 to 20 (10^5^ points in total) for a given *θ*_c_. The process is repeated for different *θ*_c_ values ranging from 0° to 90°. To visualize the degree of diversity in the transmission phase combinations, a density map is plotted on a 20 × 20 grid of the two-dimensional phase space for two representative values of *θ*_c_: 0° (Figure 3(b)) and 8° (Figure 3(c)). The map shows the number of realized phase combinations within each grid block with the degree of shading on a logarithmic scale. Based on the density map, the filling ratio can be defined as the number of grid blocks with nonzero counts divided by the total number of blocks. We note that cases with poor transmittance ( ${\left|t\right|}^{2}<0.5$ ) are rejected in the counts for Figure 3(a)–(c). The highest filling ratio is 87% at *θ*_c_ = 8°, which is more than twice as large as 42% at *θ*_c_ = 0° (i.e., vertical crystal axis), as shown in Figure 3(a). Only 13% of the phase space is missed with *θ*_c_ = 8°, while 58% of the blocks are unreachable with *θ*_c_ = 0°. The analytical investigation shows that an anisotropic medium with a properly slanted crystal axis facilitates high-efficiency, independent transmission phase control for input beams with near-zero incidence angles.

Such a vivid difference between the paraxial performances of anisotropic crystals with a vertically aligned and optimally slanted optical axis can be understood if we look at their complex transmission coefficients. The transmission coefficients for two incident angles, denoted by different markers, are plotted as a function of ϵ_3_ in Figure 3(d)–(f). Here, we focus on small values of ϵ_3_ because large phase differences are typically achieved under such conditions (Supporting Information) and fix ϵ_1_ to an intermediary value of 10 as a representative example. For the vertical crystal axis (Figure 3(d)), the transmission does not depend on ϵ_3_ at a normal incidence angle (*θ*_1_ = 0°) as the incident wave does not have vertical electric components, and all data points coincide. With an oblique incidence angle (*θ*_2_ = 10°), the transmissions follow a peanut-like trajectory on the complex plane. Even though the anisotropic medium with a vertical crystal axis can induce large phase differences between two incident angles at some ϵ_3_ values, most of those points have transmission amplitudes smaller than the threshold ( ${\left|t\right|}^{2}=0.5$ ), which is denoted by the dashed circle. The corresponding phase difference is plotted as a line graph in Figure 3(g), with shaded regions indicating where transmittance is low for at least one of the incident angles. In contrast, a properly inclined angle of the crystal axis enables a high-efficiency, large phase gradient for inputs with a near-zero incidence angle. The phase difference between two inputs can reach ±*π* while maintaining transmission amplitudes above the threshold (Figure 3(e) and (h)). However, not all the inclined angles of the crystal axis satisfy both high-efficiency transmittance and a large phase gradient. For example, as shown in Figure 3(f) and (i), although they have high transmittance values over a broad range of ϵ_3_ with a 45-degree slating of the crystal axis, the phase differences between the two inputs are small and less sensitive to ϵ_3_ values.

The physical origin of these phenomena can be found in the dependence of the refractive index and the wave impedance on the incident angle and ϵ_3_. For the anisotropic medium with a vertical crystal axis, the derivative of the refractive index with respect to the incidence angle significantly increases as the ϵ_3_ reduces to very small values, which signals that large phase differences would be possible for small incident angle changes. However, at the same time, the wave impedance and its derivative also dramatically increase, making it difficult to achieve the impedance matching required for high transmittance unless the Fabry–Pérot resonance condition is met (Supporting Information). With the tilted crystal axes, the wave impedance and its derivative are decoupled from the refractive index and impedance matching can be approximately maintained over a wide range of incident angles and ϵ_3_ values, enabling higher transmission efficiencies. Moreover, with nonzero *θ*_c_, the phase difference is maximized at finite ϵ_1_ and nonzero ϵ_3_ values (Supporting Information), making the optimal configuration practically easier to achieve compared to the vertical case, which requires smaller ϵ_3_ values.

From the analysis of the anisotropic effective medium model, we conclude that a properly slanted optical axis enables high angular sensitivity of the transmission phases for paraxial rays propagating through an anisotropic slab while retaining high transmission efficiency. As a final remark of the section, we note that the effective angle of the crystal axis (*θ*_c_) does not necessarily equal the physical slanting angle (*θ*_s_), due to the finite thickness of the slab and the surface effects, and it can be found with numerical fitting.

## 3 Angle-multiplexed phase gradient metasurface

Based on the diverse combinations of angle-sensitive transmission phases discussed in the previous section, one can build angle-multiplexed or angle-selective metasurfaces with high optical efficiency by spatially arranging such elements. This section includes two examples: an angle-sensitive beam deflector and an angle-multiplexed metalens.

### 3.1 Angle-multiplexed beam deflector

The beam deflector example is designed to show the design versatility and large degree of freedom of the proposed metasurface elements. If the metasurface elements have low angular sensitivity, two incident beams separated by a small incident angle separation would acquire similar transmission phases as a function of their spatial positions and, hence, will be deflected in similar directions. In this study, we present an angle-sensitive beam deflector with high angular sensitivity that can direct two beams with similar incident angles in two very different, yet designated directions.

**Figure 4:**
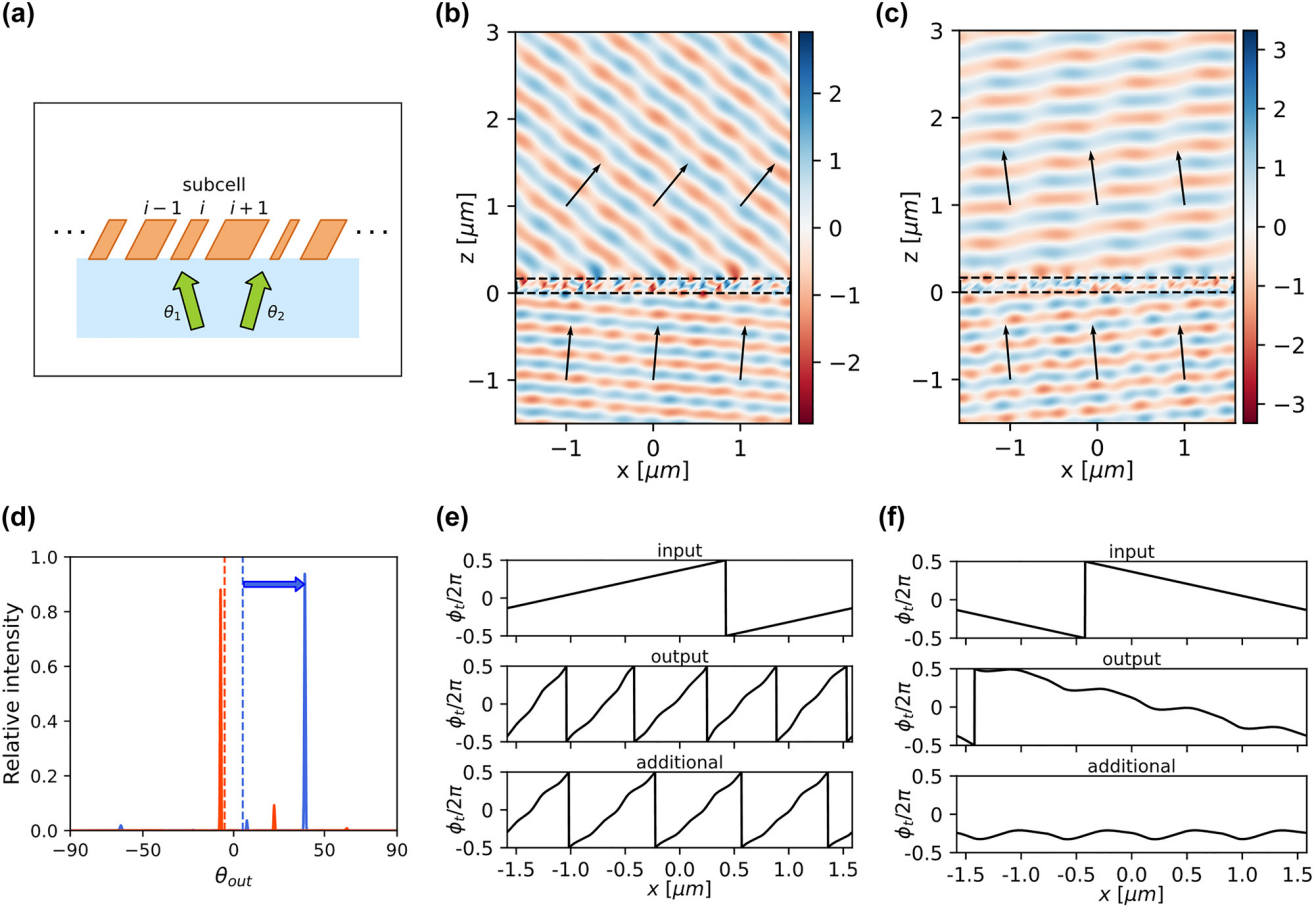
Optimized angle multiplexed beam deflector with symmetric input angles. (a) Schematic of structures for simulation. Supercell includes subcells with different width and same slanted angle and height. Input waves are in the substrate. (b) and (c) The real part of electric field phasors for the *p*-polarized incidences, (b) *θ*_1_ = +5°, (c) *θ*_2_ = −5°. (d) Diffraction efficiencies over the angle of outgoing wave. The blue and red dashed line indicates input angles with a unity intensity *θ*_1_ and *θ*_2_, respectively. The solid lines indicate the relative intensities at diffracted angle with each incidences. The desired angles are 39.2° and −7.3°, respectively. (e–f) The spatial phases of input, output, and additional phase shifts for each incidence, (e) *θ*_1_ = +5°, (f) *θ*_2_ = −5°.

To achieve this goal, the metasurface should be able to provide a completely different phase gradient profile $d\phi \left(x;\theta_{\text{i}}\right)/dx$ for each incidence angle (*θ*_i_), as shown in the following equation [27]:

(2) $$\begin{array}{c} n_{\text{t}}\sin\theta_{\text{t}}-n_{\text{i}}\sin\theta_{\text{i}}=\frac{\lambda }{2\pi }\frac{\text{d}\phi }{dx}=\frac{\lambda }{2\pi }\left(m\frac{2\pi }{\Lambda }\right), \end{array}$$

where *n*_i_ and *n*_t_ are the refractive indices of the incident and departing medium, respectively, *θ*_t_ is the departing angle, *λ* is the wavelength of the incident light, *Λ* is the supercell period, and *m* is an integer indicating the order of diffraction. Independent control of beam deflection for two incidence angles can be realized if *m* can be set differently for each incidence angle of interest. While we assumed periodicity for convenience, the formulism can also be applied to aperiodic cases if the supercell period is set to infinity. For small deflection angles, unit structures can be selected from a library (Figure 2(f)) and spatially placed based on the desired phase profile. However, due to near-field coupling between neighboring structures, the overall response of the designed metasurface can deviate from the target phase modulation, particularly in large deflection angle cases with highly dissimilar neighbors, resulting in undesirable diffraction orders. For better performance, one can adopt a hybrid inverse design method that combines gradient-based shape optimization with stochastic particle-swarm optimization (PSO) to efficiently discover both local (*w*_*i*_) and global parameters (*h*, *θ*_s_) [28] (Figure 4(a)). With random initial particle positions in terms of *h* and *θ*_s_, adjoint simulation-based shape optimization is performed to find the optimal *w*_*i*_ of each unit structure, after which the parameters *h* and *θ*_s_ are updated by PSO and the process is repeated (Supporting Information). We use the following figure of merit (FoM) for the beam deflection problem with two incident angles:

(3) $$\begin{array}{c} \text{FoM}=a_{1}T_{1}+a_{2}T_{2} \end{array}$$

where *T*_*i*_ is the calculated deflection efficiency toward the desired directions for each input beam with incident angle *θ*_*i*_ and *a*_*i*_ is a weighting factor (*i* = 1, 2).

Figure 4 shows an example of a selective beam deflector that can deflect two *p*-polarized incident beams with a small angular separation (*θ*_1_ = +5° and *θ*_2_ = −5° from the glass side) in two very different, yet designated directions (39.2° and −7.3° are chosen in this example). To achieve the target deflection angles, we optimized 12 TiO_2_ subcells on a glass substrate, constituting a periodic supercell with a period of 3170 nm. All subcells have a common optimized slanted angle and thickness (50.1° and 167 nm, respectively). The optimized width of each fin structure varies between 116 and 195 nm (Supporting Information). Figure 4(b) and (c) shows the real part of the electric field phasors for each incident wave. The profiles show clean outgoing plane waves propagating in targeted directions. The relative efficiencies (normalized by the total transmittance) of deflected beams and their additional phase shifts are plotted in Figure 4(d)–(f). The numerically measured relative deflection efficiencies to the targeted directions are high at 93.8% (absolute efficiency: 83%) and 88% (absolute efficiency: 56.3%) for +5° and −5° angles of incidence, and the phase gradients (d*ϕ*/d*x*) provided by the metasurface are 8*π*/*Λ* and 0, respectively, where *Λ* is the supercell period. This example illustrates that optimized slanted gratings enable high-efficiency selective beam deflection and can achieve a large asymmetric phase gradient even for symmetric, near-zero incidence angles, which are impossible for vertical gratings.

**Figure 5:**
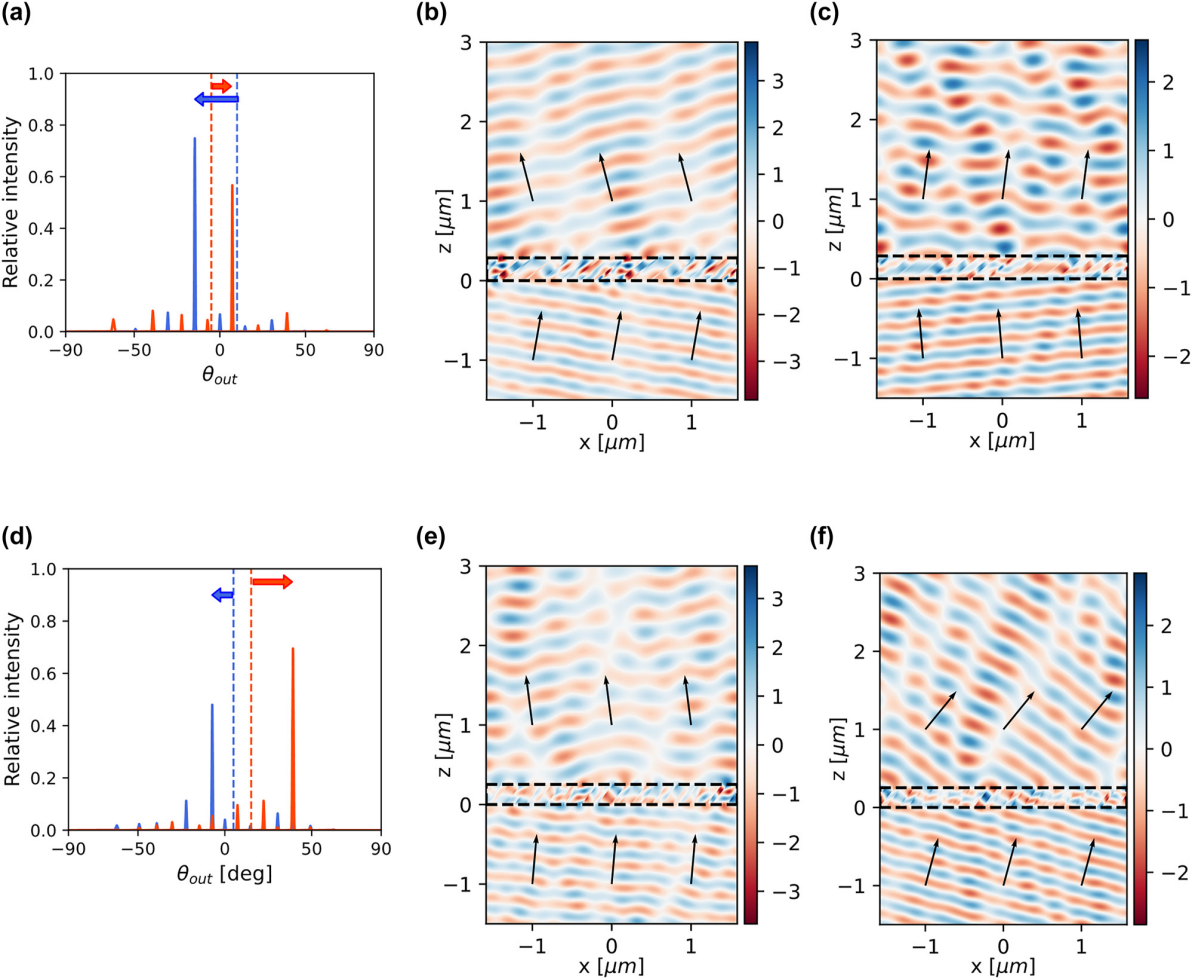
Optimized angle multiplexed beam deflector for (a)–(c) negative refraction and (d)–(f) superprism-like behavior. (a) and (d) Diffraction efficiencies over the angle of outgoing wave. (a) The blue and red dashed line indicates angles of *p*-polarized input beams with a unity intensity *θ*_1_ = +10° and *θ*_2_ = −5°, and the corresponding desired diffracted angles are −14.6° and +7.3°, respectively. (b) The blue and red dashed line indicates input angles with a unity intensity *θ*_1_ = +5° and *θ*_2_ = +15.2°, and the corresponding desired diffracted angles are −7.3° and +39.2°, respectively. (b), (c) and (e), (f) The real part of electric field phasors for the incidences, (b) *θ*_1_ = +10°, (c) *θ*_2_ = −5° and (e) *θ*_1_ = +5°, (f) *θ*_2_ = +15.2°.

The degree of freedom in design is very large: there are 14 parameters that can be tuned independently in the above example. Therefore, target input and output angle combinations can be chosen almost arbitrarily. Another example is shown in Figure 5(a)–(c), where two *p*-polarized asymmetrically incident waves (*θ*_1_ = +10° and *θ*_2_ = −5°) can both be negatively refracted to −14.6° and +7.3° with relative deflection efficiencies of 75% and 57%, respectively. It is also possible to achieve superprism-like behavior for two input angles, *θ*_1_ = +5° and *θ*_2_ = +15.2°, which are diverted to −7.3° and +39.2°, respectively, with relative efficiencies of 48% and 69.5%. The angular separation is more than four times larger than the incident angle separation. Because the supercell period (*Λ*) constrains the possible phase gradients to discrete values (integer multiples of 2*π*/Λ), *Λ* can be adjusted if other combinations of input and output angles are desired. Alternatively, an aperiodic design (i.e., infinite *Λ*) would allow for a continuous range of angles, albeit at the expense of increased design complexity. In general, allowing a nonzero slanting angle for the grating structure enables diverse angle-dependent deflection properties, which are critical for applications involving various types of beam-steering devices.

### 3.2 Angle-multiplexed metalens array

**Figure 6:**
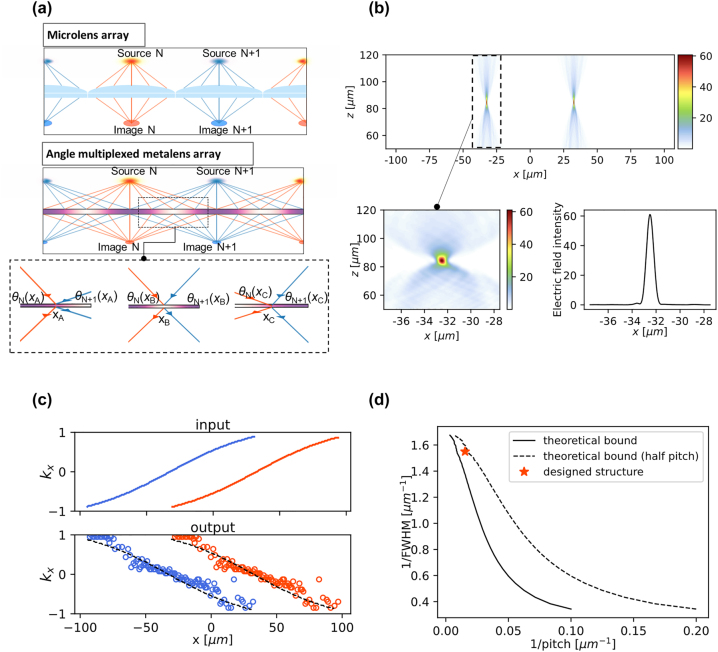
Optimized angle multiplexed metalens array. (a) Schematic of conventional microlens array (top of figure) and angle multiplexed metalens array (bottom of figure). In the magnified figure, two sets of incident angles which come from adjacent sources are considered in each different local region and are deflected in different directions. (b) The intensity profiles in the focal planes at 532 nm wavelength of the optimized metalens with 130 μm lens diameter and 65 μm pitch of hot spots. For input beams, two-dimensional *p*-polarized Gaussian beams are used. (c) The spatial distributions of tangential wavevector *k*_x_ for inputs and outputs. The *k*_x_ of incident beams from two sources are drawn with blue and orange colored curves, and *k*_x_ of the output beams are drawn with circles. The target deflection denoted with dashed lines. (d) The Pareto frontier of the conventional microlens array calculated from the analytical equation. The dashed line is the bound of performance calculated by assuming lenses having half of the pitch with the same lens diameter of conventional lenses. The optimized angle-sensitive metalens, indicated by the orange star, close to the theoretical bound.

Angle-sensitive metasurfaces can transcend the fundamental limits of conventional optical devices. As an example, we propose highly efficient angle-multiplexed metalens arrays. The asymmetric transmission characteristics of the proposed device can overcome the pitch–resolution trade-off in conventional microlens arrays, which is an important issue for imaging, optical lithography, and laser-based fabrication. In conventional devices, individual lenses in a lens array cannot physically overlap with one another. Hence, a smaller pitch between adjacent lenses, which aids in increasing the density of “hot spots”, necessarily results in a smaller diameter of each lens, which degrades resolution (larger hot spot size) owing to the loss of high spatial frequency components (top of Figure 6(a)). Previous studies have proposed advanced metalenses for high numerical apertures [29–31] as well as for correcting monochromatic [32–35] or chromatic aberrations [36–39]. However, these metalenses cannot overcome the pitch–resolution trade-off. Interleaved metalenses can partially tackle the trade-off issue, but their spatial multiplexing strategy necessarily results in proportional sacrifice in optical efficiency [40, 41]. To address this issue, we introduce an angle-multiplexed metalens array that can have a lens diameter nearly twice that of conventional lenses or metalens arrays with the same pitch while maintaining efficiency (bottom of Figure 6(a)). In many microlens array applications, in which a planar array of hot spots is imaged onto a corresponding array of spots at a sensor plane or laser output plane, a relatively small range of angle of incidence is utilized at each local area of the lens surface. These incident waves are deflected into a small range of output directions. In contrast, in the angle-multiplexed metalens array, two or more different sets of incident angles are considered in the local region (coming from two or more adjacent sources) and are deflected in different directions depending on their target image spots. Because the same location of the lens can provide different deflection angles for incident waves coming from different source directions, it can effectively realize overlapping lenses without compromising efficiency in principle.

To obtain high-efficiency focusing for a periodic array of two-dimensional *p*-polarized Gaussian beams with a pitch of 65 μm, a beam diameter of 130 μm, and a divergence angle of 39.1°, an array of convex metalenses with a diameter of 130 μm is designed by adjoint-based optimization. Owing to the periodicity and overlap of the angle-multiplexed metalens array, the repeating supercell of the metasurface is 65 μm wide, half the lens diameter. The FoM is defined as,

(4) $$\text{FoM}=\underset{x_{\text{tgt}}}{\int }{\left|E\left(x,f\right)\right|}^{2}dx$$

where *x*_tgt_ is the target hot spot region. The optimized design has 260 different fin structures at 250 nm center-to-center spacing, and each fin has a slant angle of 23.9° and a thickness of 479.4 nm. The result of hot spot generation using the optimized angle-multiplexed metalens array is shown in Figure 6(b). The distance between focused spots is 65 μm and the fullwidth at half maximum of the hot spot is 645.2 nm. The source normalized focusing efficiency within the beam width is 74.9% (Supporting Information). The spatial distributions of the tangential wavevector *k*_x_ for inputs and outputs are calculated in Figure 6(c). At every position between adjacent sources from −32.5 μm to 32.5 μm, the incident beams from two sources have well-separated *k*_x_ as drawn with blue and orange curves, and *k*_x_ of the output beams (circles) follow the targeted deflection denoted with dashed lines. To compare the performance of conventional lens arrays and angle-multiplexed metalens arrays, a Pareto frontier calculated from the analytical equation (Supporting Information) is drawn in Figure 4(d), which is a set of optimal designs (no design is better than these in both resolution and pitch). The optimized angle-sensitive metalens design, denoted by an orange star, can transcend the Pareto frontier of conventional lenses and is close to the theoretical bound of performance calculated by assuming lenses with half of the pitch and the same lens diameter as conventional lenses. As a consequence, the angle-multiplexed metalens array design can facilitate densely packed high-resolution imaging or hot spot formation, which is crucial for diverse applications.

## 4 Conclusions

We discussed the angular response of metasurfaces composed of slanted unit structures to break the mirror symmetry of vertical structures and demonstrated the performance of new types of angle-sensitive phase gradient metasurfaces. Vertical structures with effective permittivities aligned to the principal axes *x*, *y*, and *z* can achieve high angular sensitivity at near-zero incidences at the expense of transmission efficiency loss. Moreover, they cannot break the symmetry of transmission for two symmetric inputs with respect to the *z*-axis. However, the slanted structures not only have high angle sensitivity at near-zero incidence angles with high efficiency but they can also obtain asymmetric transmission for two symmetric incidence angles owing to the nonzero phase slope at near-normal incidence. This offers much more diverse phase engineering possibilities, and potential applications are not limited to the examples presented in this study. Overall, we believe that this approach can serve as a way to overcome the limitations of previous methods in angle-dependent diffractive optics and will enable the development of novel devices with unforeseen functionalities.

## Supplementary Material

Supplementary Material Details
