# Supplementary material for: Highly angle-sensitive and efficient optical metasurfaces with broken mirror symmetry
Source: Nanophotonics. 2023 Feb 13;12(13):2347–58. doi: 10.1515/nanoph-2022-0793 (PMC11501263; doi:10.1515/nanoph-2022-0793)
Supplement: Supplementary file 1 — Supplementary Material Details [file j_nanoph-2022-0793_suppl.pdf]

Supporting Information: Highly angle-sensitive and efficient optical metasurfaces with broken mirror symmetry

Nayoung Kim[1], Myungjoon Kim[1], Joonkyo Jung[1], Taeyong Chang[1], Suwan Jeon[1], and Jonghwa Shin[1]

[1]*Department of Materials Science and Engineering, Korea Advanced Institute of Science and Technology, Daejeon 34141, Republic of Korea*

CONTENTS

I. Delaunay triangulation

II. S-parameter calculation in anisotropic media

III. Angle-sensitivity analysis with permittivities

IV. Inverse design framework and flowchart

V. Focusing efficiency calculation for metalens

VI. Pareto frontier calculation for metalens

VII. Optimization with different initial parameters

VIII. Geometry parameters

I. DELAUNAY TRIANGULATION

When dividing the space by connecting points on the plane with triangles, the Delaunay triangulation is a division in which the minimum value of the interior angles of these triangles becomes the maximum. From the Delaunay triangulation, the circumcircle of any triangles contains no points other than the three vertices of the triangle. The biggest void in the phase space can be quantitively measured by conducting the Delaunay triangulation to Fig.2 in the main text and finding the maximum radius of the circumcircles.

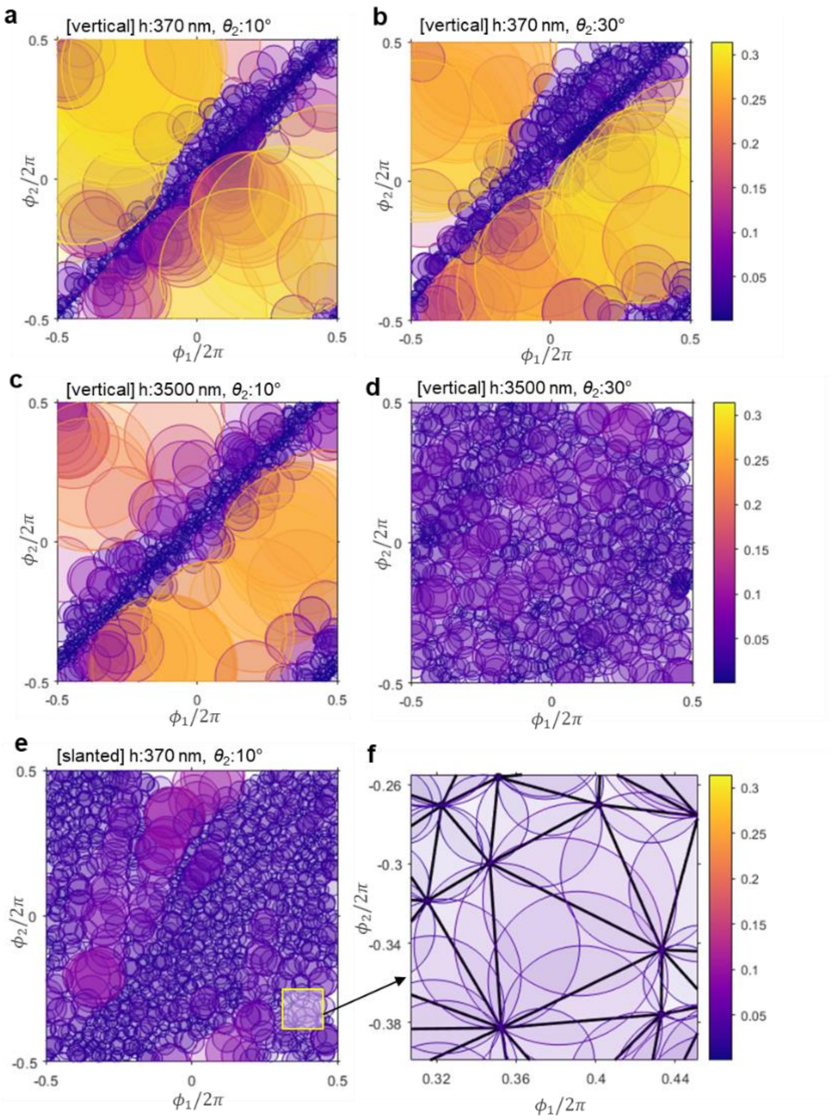

Figure S1 Delaunay triangulation of the phase space. The colors represent the radius of circumcircles. One of the incidence angles is fixed as $\theta_1 = 10^o$. (a)–(b) Circumcircles of each triangle which is the division of the phase points of vertical gratings with 370 nm thickness. (a)$\theta_2 = 10^o$, (b)$\theta_2 = 30^o$. (c)–(d) Circumcircles of each triangle which is the division of the phase points of vertical gratings with 3500 nm thickness. (c)$\theta_2 = 10^o$, (d)$\theta_2 = 30^o$. (e)–(f) Circumcircles of each triangle which is the division of the phase points of slanted gratings with 370 nm thickness and $\theta_2 = 10^o$.

II. S-PARAMETER CALCULATION IN ANISOTROPIC MEDIA

To analyze angle-sensitive response of anisotropic media with rotated in x-z plane, scattering of obliquely incident planewaves from an anisotropic slab with finite thickness *d* and infinite in the *x*- and *y*-directions is calculated [1,2]. The anisotropic constitutive relations considered are

$$\mathbf{D} = \bar{\bar{\epsilon}} \cdot \mathbf{E}$$
$$\mathbf{B} = \bar{\bar{\mu}} \cdot \mathbf{H} \tag{S1}$$

where

$$\bar{\bar{\epsilon}} = \epsilon_0 \begin{pmatrix} \epsilon_x & 0 & \epsilon_{xz} \\ 0 & \epsilon_y & 0 \\ \epsilon_{xz} & 0 & \epsilon_z \end{pmatrix} = \epsilon_0 \begin{pmatrix} \cos\theta_c & 0 & \sin\theta_c \\ 0 & 1 & 0 \\ -\sin\theta_c & 0 & \cos\theta_c \end{pmatrix} \begin{pmatrix} \epsilon_1 & 0 & 0 \\ 0 & \epsilon_2 & 0 \\ 0 & 0 & \epsilon_3 \end{pmatrix} \begin{pmatrix} \cos\theta_c & 0 & \sin\theta_c \\ 0 & 1 & 0 \\ -\sin\theta_c & 0 & \cos\theta_c \end{pmatrix}$$

$$\bar{\bar{\mu}} = \mu_0 \begin{pmatrix} \mu_x & 0 & 0 \\ 0 & \mu_y & 0 \\ 0 & 0 & \mu_z \end{pmatrix}$$

where ϵ_0 and μ_0 are the permittivity and permeability of free space.

Following the reference [1,2], the state equation defining the wave propagation within the anisotropic medium is:

$$\frac{\mathrm{d}}{\mathrm{dz}} \begin{bmatrix} E_s \\ H_s \end{bmatrix} = \bar{\bar{\Gamma}} \begin{bmatrix} E_s \\ H_s \end{bmatrix} \tag{S2}$$

where E_s and H_s are the transverse electromagnetic components and $\bar{\bar{\Gamma}}_{ij}$ can be calculated from [1,2]. The state vectors at the slab's boundaries *z* = 0, *d* have a relation as

$$\bar{\psi}(0) = \bar{\bar{A}} \bar{\psi}(d) \tag{S3}$$

where $\bar{\bar{A}} = \exp(-\bar{\bar{\Gamma}}\, d)$ and $\bar{\psi} = [E_s, H_s]$. By solving the eigenvalue problem $\bar{\bar{A}} = \bar{\bar{V}}\bar{\bar{\Lambda}}\bar{\bar{V}}^{-1}$, we can obtain wave impedance and propagation vector in anisotropic media.

$$\eta = \frac{\sqrt{\epsilon_z \mu_y}}{\sqrt{\epsilon_x \epsilon_z - \epsilon_{xz}^2}} \tag{S4}$$

$$k_z = \frac{k_0}{\epsilon_z}\left(-\epsilon_{xz}\, sin\, \theta_i + \sqrt{(\epsilon_x \epsilon_z - \epsilon_{xz}^2)(\epsilon_z \mu_y - \sin^2\theta_i)}\right) \tag{S5}$$

Therefore, the reflection and transmission coefficients for p-polarization can be obtained as [1,2]

$$\mathrm{S}_{11} = \frac{\Gamma(1 - e^{i2k_z d})}{1 - \Gamma^2 e^{i2k_z d}}$$

$$\mathrm{S}_{21} = \frac{(1 - \Gamma^2) e^{ik_z d}}{1 - \Gamma^2 e^{i2k_z d}} \tag{S6}$$

where $\Gamma = \frac{\eta/\cos\theta_i - 1}{\eta/\cos\theta_i + 1}$ for incidence angle θ_i.

III. ANGLE-SENSITIVITY ANALYSIS WITH PERMITTIVITIES

The small extraordinary permittivity values plays an important role in achieving a large phase delay between the two inputs with near-zero incidence angles as shown in Fig.3 in main text. The reason can be graphically illustrated with equi-frequency contour of anisotropic media. The dispersion relation of anisotropic media can be calculated as [3]:

$$\bar{\bar{M}} = (k \times \bar{\bar{I}})\bar{\bar{\mu}}^{-1}(k \times \bar{\bar{I}}) + \omega^2 \bar{\bar{\epsilon}} \tag{S7}$$

For the existence of nontrivial solutions, $\det(\bar{\bar{M}})$ =0. This gives the dispersion relation as follows.

$$\epsilon_{yy}\mu_{xx}\mu_{zz}k_0^2 = \mu_{xx}k_x^2 + \mu_{zz}k_z^2 \tag{S8a}$$

$$(\epsilon_{xx}\epsilon_{zz}\mu_{yy} - \epsilon_{xz}^2\mu_{yy})k_0^2 = \epsilon_{xx}k_x^2 + 2\epsilon_{xz}k_xk_z + \epsilon_{zz}k_z^2 \tag{S8b}$$

where the notation of constitutive parameters is represented in eq.1 in the main text.

Equation S8a represents the dispersion relation of *s*-polarized plane waves and eq.S8b represents the dispersion relation of *p*-polarized plane waves. For p-polarized waves, eq.S8b can be represented with permittivities in principal dielectric axes.

$$\left(\frac{\cos\theta_c^2}{\epsilon_3} + \frac{\sin\theta_c^2}{\epsilon_1}\right)k_x^2 + 2\left(\frac{1}{\epsilon_1} - \frac{1}{\epsilon_3}\right)k_xk_z\sin\theta_c\cos\theta_c + \left(\frac{\cos\theta_c^2}{\epsilon_1} + \frac{\sin\theta_c^2}{\epsilon_3}\right)k_z^2 = k_0^2\mu_y \tag{S9}$$

Figure S2 graphically shows the equi-frequency contour and difference of propagation wavenumber (Δk_z) for two inputs as a function of angle of crystal axis (θ_c). Using a value of ϵ_3 close to zero can yield large Δk_z, especially when $\theta_c = 0$. However, with larger ϵ_3 values, Δk_z decreases significantly over the entire range of θ_c, and a maximum Δk_z value can be achieved at a specific θ_c.

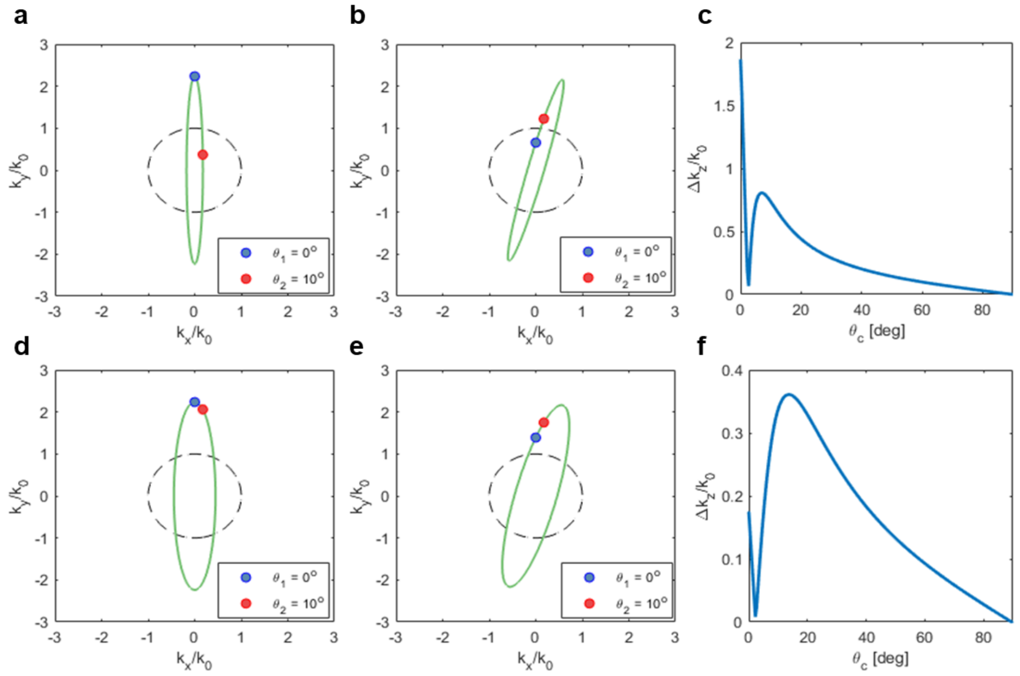

Figure S2 Equi-frequency contour (EFC) of anisotropic media and difference of propagation vector. Permittivity in x-direction is fixed (ϵ_1=5). (a)–(c) EFC and Δk_z for anisotropic media with ϵ_3=0.031. (a)$\theta_c = 0^o$, (b)$\theta_c = 15^o$, (c)Δk_z as a function of θ_c. (d)–(f) EFC and Δk_z for anisotropic media with ϵ_3=0.2. (a)$\theta_c = 0^o$, (b)$\theta_c = 15^o$, (c)Δk_z as a function of θ_c. Red and blue circle markers indicate two different inputs.

From eq.S8b, k_z with incidence angle θ_i can be represented as

$$k_z = \frac{k_0}{\epsilon_z}\left(-\epsilon_{xz}\sin\theta_i + \sqrt{(\epsilon_x\epsilon_z - \epsilon_{xz}^2)(\epsilon_z\mu_y - \sin^2\theta_i)}\right) \tag{S10}$$

The Δk_z for two inputs with incidence angle θ_1 and θ_2 is,

$$\Delta k_z = k_{z2} - k_{z1} = \frac{k_0}{\epsilon_z}\left(-\epsilon_{xz}(\sin\theta_2 - \sin\theta_1) + \sqrt{\epsilon_x\epsilon_z - \epsilon_{xz}^2}\left(\sqrt{\epsilon_z\mu_y - \sin^2\theta_2} - \sqrt{\epsilon_z\mu_y - \sin^2\theta_1}\right)\right) \tag{S11}$$

For small θ_1 and θ_2,

$$\Delta k_z \cong \frac{k_0}{\epsilon_z}\left(-\epsilon_{xz}(\sin\theta_2 - \sin\theta_1) + \sqrt{\epsilon_x\epsilon_z - \epsilon_{xz}^2}\left(\sqrt{\epsilon_z\mu_y} - \frac{\sin^2\theta_2}{2\sqrt{\epsilon_z\mu_y}} - \left(\sqrt{\epsilon_z\mu_y} - \frac{\sin^2\theta_1}{2\sqrt{\epsilon_z\mu_y}}\right)\right)\right) \tag{S12}$$

$$\frac{\Delta k_z}{k_0} \cong \frac{-\Delta\theta}{\epsilon_z}\left(\epsilon_{xz} + \frac{\theta_{\text{center}}}{\sqrt{\epsilon_z\mu_y}}\sqrt{\epsilon_x\epsilon_z - \epsilon_{xz}^2}\right) \tag{S13}$$

where $\Delta\theta = \theta_2 - \theta_1$ and $\theta_{\text{center}} = (\theta_1 + \theta_2)/\,2$.

Note that for symmetric incidences ($\theta_{\text{center}} = 0$), ϵ_{xz} should be non-zero to achieve phase difference ($\Delta k_z \neq 0$).

For the simplicity of the analysis, we can ignore second order term of incidence angle (θ^2).

$$\begin{aligned}\frac{\Delta \mathrm{k_z}}{k_0} &= -\frac{\Delta\theta\cdot\theta_{\text{center}}\sqrt{\epsilon_1\epsilon_3}}{\sqrt{\mu_y}(\epsilon_3\cos^2\theta_c + \epsilon_1\sin^2\theta_c)^{3/2}} - \frac{\Delta\theta(\epsilon_3 - \epsilon_1)\cos\theta_c\sin\theta_c}{\epsilon_3\cos^2\theta_c + \epsilon_1\sin^2\theta_c} \\ &\cong -\frac{\Delta\theta(\epsilon_3 - \epsilon_1)\cos\theta_c\sin\theta_c}{\epsilon_3\cos^2\theta_c + \epsilon_1\sin^2\theta_c} = -\Delta\theta\frac{\left(\frac{\epsilon_3 - \epsilon_1}{\epsilon_3 + \epsilon_1}\right)\sin 2\theta_c}{1 + \frac{(\epsilon_3 - \epsilon_1)}{(\epsilon_3 + \epsilon_1)}\cos 2\theta_c}\end{aligned} \tag{S14}$$

$\Delta \mathrm{k_z}/k_0$ is maximized when $\epsilon_1 \to \infty$ or $\epsilon_3 \to 0$ regardless of angle of crystal axis θ_c, and the maximum value is $\cot\theta_c$. However, if the permittivities are not in global maximum, the θ_c becomes critical to obtain larger $\Delta \mathrm{k_z}$. When the ϵ_1 has finite value and ϵ_3 is non-zero, the $\Delta \mathrm{k_z}$ is maximize when $\theta_c = \arctan\left(\sqrt{\epsilon_3/\epsilon_1}\right)$. Therefore, to achieve large transmission phase difference with respect to small angle difference of two inputs, appropriate tilted crystal axis should be required.

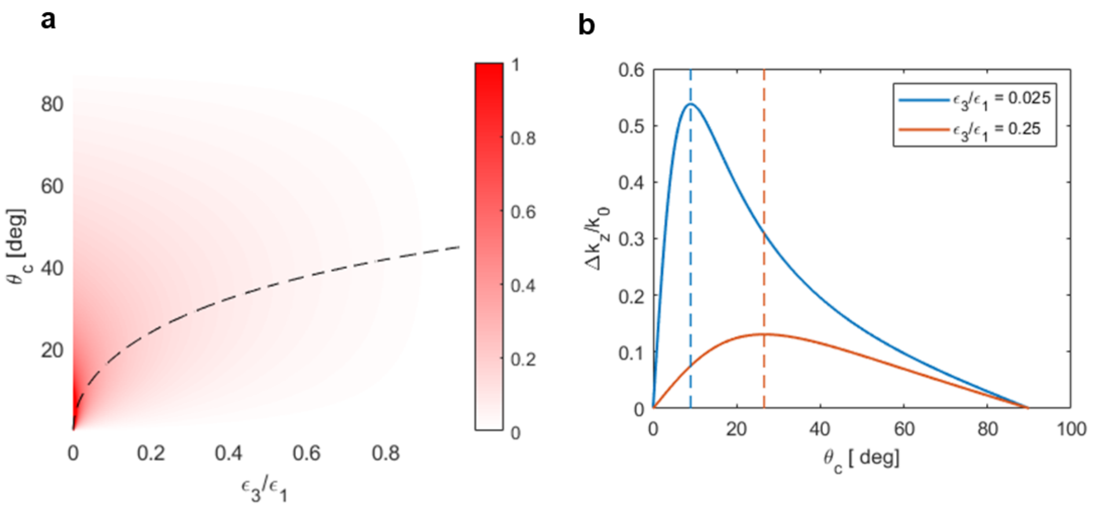

Figure S3 Propagation wavenumber difference ($\Delta k_z/k_0$) between two incidence angles $\theta_1 = 0^{\circ}$ and $\theta_2 = 10^{\circ}$.

Moreover, although the materials with vertical crystal axis can also have large phase difference between two near-zero incidences when ϵ_3 is almost near-zero, it is difficult to achieve high transmission efficiency. For the *p*-polarized incidence, the material refractive index and wave impedance with respect to incidence angle θ_i can be calculated as

$$n = \sqrt{\epsilon_{xx}\mu_{yy} + \left(1 - \frac{\epsilon_{xx}}{\epsilon_{zz}}\right)\sin^2\theta_i} \tag{S15a}$$

$$\eta = \frac{\sqrt{\mu_{yy} - \frac{\sin^2\theta_i}{\epsilon_{zz}}}}{\sqrt{\epsilon_{xx}}} \tag{S15b}$$

From the eq.S15, the refractive index derivative with respect to incidence angle, $\mathrm{d}n/\mathrm{d}\theta_i$, becomes infinite when the ϵ_{zz} (for vertical crystal axis material, $\epsilon_{zz} = \epsilon_3$) close to the zero. But at the same time, the wave impedance and its derivative become also diverges which makes it difficult to achieve high transmission efficiency except when they satisfy the Fabry–Pérot condition.

For the material with tilted crystal axis ($\epsilon_{xz} \neq 0$) the material refractive index and wave impedance with respect to incidence angle θ_i can be calculated as

$$n = \sqrt{\sin^2\theta_i + \frac{1}{\epsilon_z^2}\left(-\epsilon_{xz}\sin\theta_i + \sqrt{\epsilon_{xz}^2\left(\sin^2\theta_i - \epsilon_z\mu_y\right) + \epsilon_z\left(-\epsilon_x\sin^2\theta_i + \epsilon_x\epsilon_z\mu_y\right)}\right)^2} \tag{S16a}$$

$$\eta = \frac{\sqrt{-\sin^2\theta_i + \epsilon_z\mu_y}}{\sqrt{\epsilon_x\epsilon_z - \epsilon_{xz}^2}} \tag{S16b}$$

From the eq.S16, the refractive index derivative with respect to incidence angle becomes also infinite when the ϵ_{zz} close to the zero. However, the wave impedance and its derivative have a finite value which enable to achieve higher transmission efficiency compared to material with vertical crystal axis (eq.S17).

$$\lim_{\epsilon_{zz}\to 0} \eta = \frac{\sin\theta_i}{\epsilon_{xz}} \tag{S17a}$$

$$\lim_{\epsilon_{zz}\to 0} d\eta/d\theta_i = \frac{\cot\theta_i \sin\theta_i}{\epsilon_{xz}} \tag{S17b}$$

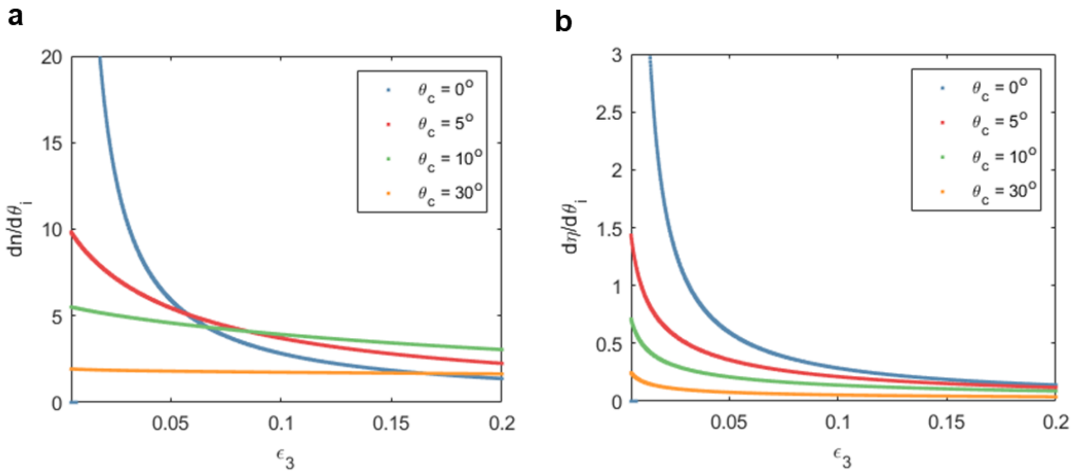

Figure S4 Derivative of refractive index and derivative of wave impedance with fixed $\theta_i = 5^\circ$ and $\epsilon_1 = 10$.

IV. INVERSE DESIGN FRAMEWORK AND FLOWCHART

In this work we design dielectric metasurfaces, optimized to perform abnormal beam deflection and imaging. The structures are consisted with slanted grating subcells with subwavelength period. For the widths of each gratings ($\boldsymbol{w}$), which is a local parameter, we adopt the adjoint-based shape optimization. The figure of merit (FoM) is defined as maximizing transmission efficiency in the target direction for beam deflector and maximizing field intensity at the focal point for metalens. The both FoM can be mathematically formulated with overlap integral obtained from Poynting vectors [4].

$$\mathrm{FoM} = \frac{1}{8}\frac{\left|\int\left[\boldsymbol{E}(\boldsymbol{r})\times\overline{\boldsymbol{H}_m(\boldsymbol{r})}+\overline{\boldsymbol{E}_m(\boldsymbol{r})}\times\boldsymbol{H}(\boldsymbol{r})\right]\cdot\mathbf{n}\,d\boldsymbol{r}\right|^2}{\int Re\left(\boldsymbol{E}_m(\boldsymbol{r})\times\overline{\boldsymbol{H}_m(r)}\right)\cdot\boldsymbol{n}\,d\boldsymbol{r}} \tag{S10}$$

where $\boldsymbol{E}_m$ and $\boldsymbol{H}_m$ represent the fields in desired direction for the beam deflector design and the field profiles of the fundamental mode at focal point for the metalens design. The overlap integral range is the supercell period for beam deflector designs and the beam width at the focal point for metalens designs. If there is a small perturbation at location $\boldsymbol{r}'$,

$$\Delta\mathrm{FoM} = \mathrm{Re}\left(A\int\left[\Delta\boldsymbol{E}(\boldsymbol{r})\times\overline{\boldsymbol{H}_m(\boldsymbol{r})}-\overline{\boldsymbol{E}_m(\boldsymbol{r})}\times\Delta\boldsymbol{H}(\boldsymbol{r})\right]\cdot\mathbf{n}\,d\boldsymbol{r}\right) \tag{S11}$$

where

$$A = \frac{1}{4}\frac{\overline{\left(\int\left[\boldsymbol{E}(\boldsymbol{r})\times\overline{\boldsymbol{H}_m(\boldsymbol{r})}+\overline{\boldsymbol{E}_m(\boldsymbol{r})}\times\boldsymbol{H}(\boldsymbol{r})\right]\cdot\mathbf{n}\,d\boldsymbol{r}\right)}}{\int Re\left(\boldsymbol{E}_m(\boldsymbol{r})\times\overline{\boldsymbol{H}_m(r)}\right)\cdot\boldsymbol{n}\,d\boldsymbol{r}} \tag{S12}$$

Using the dipole approximation, the perturbed electric and magnetic fields can be written as

$$\Delta\boldsymbol{E} = \omega^2\epsilon_0\Delta\epsilon\Delta v\boldsymbol{G_e}(\boldsymbol{r},\boldsymbol{r}')\boldsymbol{E}^f, \qquad \Delta\boldsymbol{H} = \omega^2\epsilon_0\Delta\epsilon\Delta v\boldsymbol{G_h}(\boldsymbol{r},\boldsymbol{r}')\boldsymbol{E}^f \tag{S13}$$

The gradient (eq.S11) can be reorganized using symmetry and the reciprocity.

$$\begin{aligned}\Delta\mathrm{FoM} &= \omega^2\epsilon_0\Delta v\Delta\epsilon(\boldsymbol{r}')\mathrm{Re}\left(A\int\left[\left(\boldsymbol{G_e}(\boldsymbol{r},\boldsymbol{r}')\boldsymbol{E}^f(\boldsymbol{r}')\right)\times\bar{\boldsymbol{H}}_m(\boldsymbol{r})-\bar{\boldsymbol{E}}_m(\boldsymbol{r})\times\left(\boldsymbol{G_h}(\boldsymbol{r},\boldsymbol{r}')\boldsymbol{E}^f(\boldsymbol{r}')\right)\right]\cdot\boldsymbol{n}\,d\boldsymbol{r}\right)\\ &= \omega^2\epsilon_0\Delta v\Delta\epsilon(\boldsymbol{r}')\mathrm{Re}\left(\boldsymbol{E}^f(\boldsymbol{r}')\cdot A\int\left[\boldsymbol{G_e}(\boldsymbol{r}',\boldsymbol{r})\overline{\boldsymbol{H}_m}(\boldsymbol{r})\times\boldsymbol{n}-\boldsymbol{G_h}(\boldsymbol{r}',\boldsymbol{r})\left(\boldsymbol{n}\times\overline{\boldsymbol{E}_m(\boldsymbol{r})}\right)\right]\cdot\mathbf{n}\,d\boldsymbol{r}\right)\\ &= \omega^2\epsilon_0\Delta v\Delta\epsilon(\boldsymbol{r}')\mathrm{Re}\left(\boldsymbol{E}^f(\boldsymbol{r}')\cdot\boldsymbol{E}_m^{adj}(\boldsymbol{r}')\right)\end{aligned} \tag{S14}$$

where the adjoint field is defined as:

$$\boldsymbol{E}_m^{\mathrm{adj}} = A\int\left[\boldsymbol{G_e}(\boldsymbol{r}',\boldsymbol{r})\overline{\boldsymbol{H}_m}(\boldsymbol{r})\times\boldsymbol{n}-\boldsymbol{G_h}(\boldsymbol{r}',\boldsymbol{r})\left(\boldsymbol{n}\times\overline{\boldsymbol{E}_m(\boldsymbol{r})}\right)\right]\cdot\mathbf{n}\,d\boldsymbol{r} \tag{S15}$$

The beam deflection designs are optimized using inverse design method that combines of adjoint-based shape optimization with particle-swarm optimization. With initial random particles of global parameters (h, θ_c), adjoint-based shape optimization is conducted to find optimal local parameter (w_i) of each subcell. The flow chart of the inverse design is illustrated in Fig.S3.

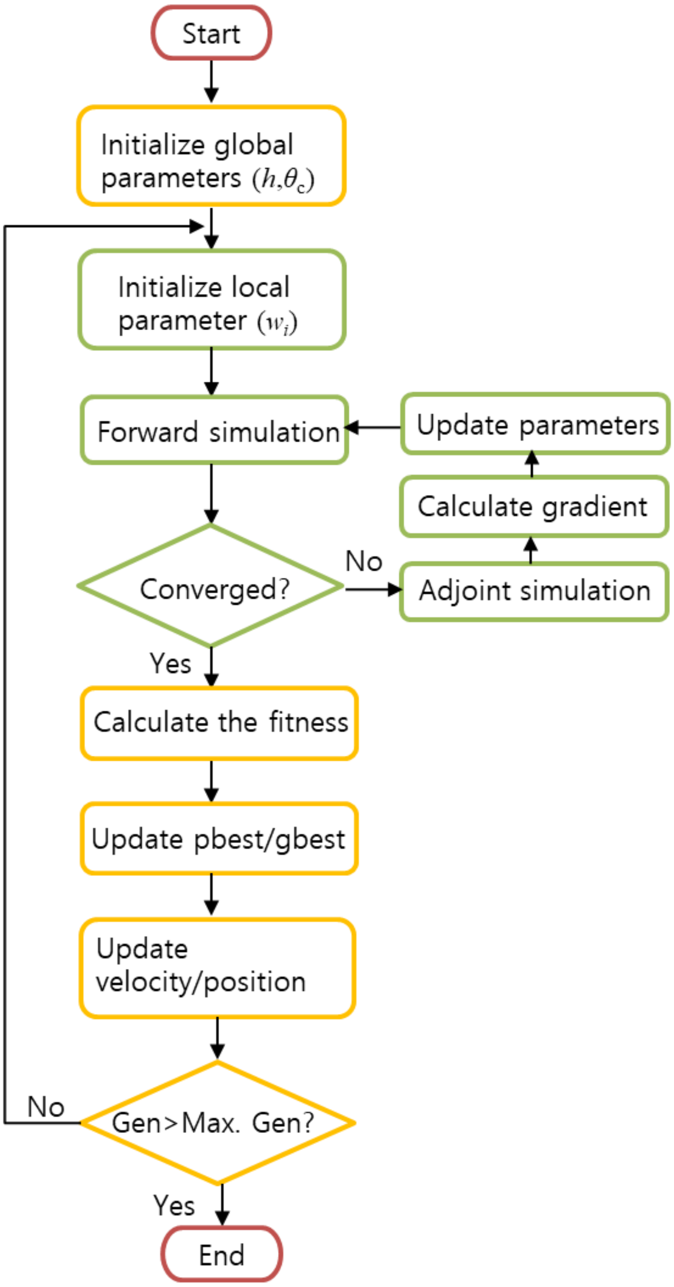

Figure S3 Flow chart of the iterative optimization.

V. FOCUSING EFFICIENCY CALCULATION FOR METALENS

The focusing efficiency is calculated by integrating of Poynting vector in focal plane.

$$P = \frac{1}{2}\int Re(E \times H^{*})\, dx \tag{S16}$$

The integral is performed within bandwidth of focused beam, which is defined the width at $1/e^2$ of maximum intensity. The focused efficiency is 66.5% (source normalized efficiency is 74.9%) for the metalens with 130 μm lens diameter.

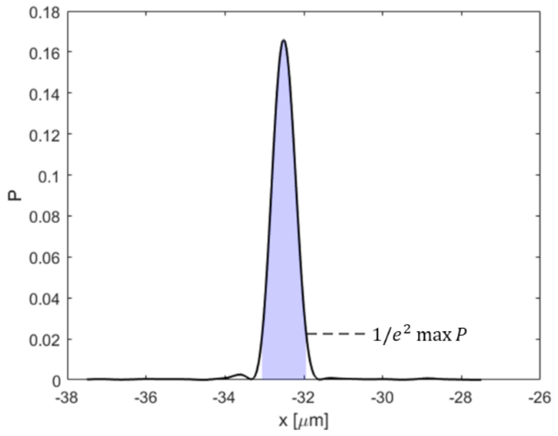

Figure S4 Focused efficiency calculation with Poynting vector at focal plane.

VI. PARETO FRONTIER CALCULATION FOR METALENS

The Pareto Frontier with respect to bandwidth and pitch (Fig.6d) is analytically calculated by Gaussian beam propagation. The electric field at source plane is defined as

$$\boldsymbol{E}(\boldsymbol{r}, z = z_s) = A_0 \exp\left(-\frac{\boldsymbol{r}^2}{w_0^2}\right) \tag{S17}$$

At the location of lens,

$$\boldsymbol{E}(\boldsymbol{r}, z = z_l) = A_0 \left(\frac{w_0}{w_l}\right) \exp\left(-\frac{\boldsymbol{r}^2}{w_l^2}\right) \exp\left(-i\left(kz_l + \frac{k\boldsymbol{r}^2}{2R_l} - \xi_l\right)\right) \tag{S18}$$

where w_l, R_l, and ξ_l are beam radius, radius of curvature of the beam's wavefront, and Gouy phase at axial distance z_l, respectively. When the source transmits through the lens with finite diameter D, we assume that the field is truncated with window function, and phase is inversed. The field is expressed as

$$\boldsymbol{E}'(\boldsymbol{r}, z = z_l) = A_0 \left(\frac{w_0}{w_l}\right) \exp\left(-\frac{\boldsymbol{r}^2}{w_l^2}\right) H(\boldsymbol{r}) \exp\left(i\left(kz_l + \frac{k\boldsymbol{r}^2}{2R_l} - \xi_l\right)\right) \tag{S19}$$

The window function is defined as

$$H(\boldsymbol{r}) = \begin{cases} 1, & -\frac{D}{2} \leq \boldsymbol{r} < \frac{D}{2} \\ 0, & otherwise \end{cases} \tag{S20}$$

In order to calculate the propagation of the field in eq.S19, we convert the field to the frequency domain by performing the Fourier transform.

$$\boldsymbol{E}'(\boldsymbol{r}, z = z_l) \xrightarrow{\mathcal{F}} \boldsymbol{E}'(\boldsymbol{k}_r, z = z_l) \tag{S21}$$

When the field propagates the distance L, it can be formulated as

$$\boldsymbol{E}'(\boldsymbol{k}_r, z = z_l + L) = \boldsymbol{E}'(\boldsymbol{k}_r, z = z_l) \exp(i\boldsymbol{k}_z L) \tag{S22}$$

Finally, the field at the distance L in the spatial domain can be obtained by inverse Fourier transform.

$$\boldsymbol{E}'(\boldsymbol{k}_r, z = z_l + L) \xrightarrow{\mathcal{F}^{-1}} \boldsymbol{E}'(\boldsymbol{r}, z = z_l + L) \tag{S23}$$

VII. OPTIMIZATION WITH DIFFERENT INITIAL PARAMETERS

In the optimization with slanted gratings, the parameters can be divided as local (w_i) and global parameters (h, θ_s). The reason for adopting inverse design method that combines of adjoint-based shape optimization with particle-swarm optimization is that global parameters make the problem to be ill-conditioned where small changes in the initial parameters can change the output, which leads to slow convergence. Table S1-3 shows the effect of each initial parameter on the optimized efficiency and structures. The small change of initial parameters of h and θ_s give prominently different optimized efficiency and structures, which illustrate the problem with the parameters is ill-conditioned. Therefore, the combines of adjoint-based local-optimization design with a global-optimization can more efficiently discover both local and global parameters for the optimization problem with slanted grating structures.

Table S1. Optimized results with different initial θ_s

θ_s	Results													
	w_i/p												T_1	T_2
40°	0.455	0.678	0.442	0.703	0.457	0.639	0.468	0.679	0.455	0.722	0.436	0.699	0.437	0.488
45°	0.368	0.724	0.367	0.661	0.426	0.713	0.410	0.724	0.403	0.633	0.441	0.737	0.385	0.441
50°	0.228	0.752	0.273	0.514	0.259	0.642	0.375	0.646	0.388	0.647	0.377	0.626	0.334	0.358

Table S2. Optimized results with different initial h

h [nm]	Results													
	w_i/p												T_1	T_2
155	0.416	0.756	0.397	0.759	0.493	0.706	0.365	0.314	0.497	0.809	0.431	0.755	0.158	0.542
166.7	0.455	0.678	0.442	0.703	0.457	0.639	0.468	0.679	0.455	0.722	0.436	0.699	0.437	0.488
175	0.432	0.215	0.498	0.689	0.456	0.661	0.444	0.656	0.436	0.674	0.427	0.727	0.313	0.490

Table S3. Optimized results with different initial w_6

w_6/p p=264 nm	Results													
	w_i/p												T_1	T_2
0.5	0.455	0.678	0.442	0.703	0.457	0.639	0.468	0.679	0.455	0.722	0.436	0.699	0.437	0.488
0.6	0.440	0.691	0.449	0.656	0.465	0.701	0.453	0.668	0.456	0.721	0.447	0.710	0.416	0.492
0.8	0.443	0.697	0.445	0.693	0.442	0.710	0.462	0.644	0.455	0.692	0.450	0.713	0.417	0.494

VIII. GEOMETRY PARAMETERS

Table S4. Optimized geometry parameters of angle-sensitive beam deflector

	w_1/p	w_2/p	w_3/p	w_4/p	w_5/p	w_6/p	w_7/p	w_8/p	w_9/p	w_{10}/p	w_{11}/p	w_{12}/p	$\boldsymbol{h}$	$\boldsymbol{\theta_c}$
Fig.4	0.417	0.512	0.732	0.415	0.509	0.732	0.418	0.511	0.732	0.417	0.511	0.735	166.7 nm	50°
Fig.5a-c	0.896	0.100	0.100	0.480	0.552	0.687	0.898	0.100	0.100	0.480	0.547	0.690	286.4 nm	52°
Fig.5d-f	0.318	0.409	0.479	0.501	0.834	0.310	0.358	0.429	0.470	0.488	0.763	0.715	253.1 nm	39°

Table S5. Optimized geometry parameters of angle-multiplexed metalens

i	w_i/p	i	w_i/p	i	w_i/p	i	w_i/p	i	w_i/p
1	0.6194	54	0.6189	107	0.5242	160	0.4315	213	0.5612
2	0.4888	55	0.5207	108	0.4280	161	0.5617	214	0.6823
3	0.4894	56	0.4738	109	0.5375	162	0.2912	215	0.2020
4	0.5014	57	0.3763	110	0.6077	163	0.4742	216	0.3014
5	0.5017	58	0.2677	111	0.4310	164	0.5301	217	0.4034
6	0.4976	59	0.6504	112	0.7112	165	0.6113	218	0.4594
7	0.5005	60	0.5431	113	0.4873	166	0.4068	219	0.5295
8	0.4919	61	0.4893	114	0.3668	167	0.5327	220	0.5444
9	0.4792	62	0.4066	115	0.5283	168	0.1476	221	0.6668
10	0.4748	63	0.2863	116	0.5462	169	0.4734	222	0.7256
11	0.4710	64	0.6251	117	0.3845	170	0.4986	223	0.3414
12	0.4550	65	0.5160	118	0.5510	171	0.6243	224	0.3927
13	0.4346	66	0.4470	119	0.4162	172	0.3593	225	0.4461
14	0.4121	67	0.7900	120	0.5746	173	0.4797	226	0.4790
15	0.4042	68	0.1826	121	0.5021	174	0.6522	227	0.6101
16	0.3820	69	0.5861	122	0.5005	175	0.1833	228	0.6202
17	0.3505	70	0.4665	123	0.5993	176	0.4184	229	0.5894
18	0.2740	71	0.3936	124	0.4341	177	0.5091	230	0.7228
19	0.2817	72	0.7651	125	0.5758	178	0.1018	231	0.7693
20	0.1778	73	0.6430	126	0.4722	179	0.4101	232	0.3297
21	0.6973	74	0.4690	127	0.5501	180	0.4937	233	0.3753
22	0.6893	75	0.3869	128	0.1978	181	0.5767	234	0.4145
23	0.6422	76	0.7551	129	0.4820	182	0.7008	235	0.4638
24	0.5571	77	0.6843	130	0.2837	183	0.3528	236	0.4922
25	0.5318	78	0.5103	131	0.4766	184	0.4989	237	0.5207
26	0.5032	79	0.3991	132	0.7276	185	0.6645	238	0.5254
27	0.4753	80	0.6892	133	0.4912	186	0.7473	239	0.5900
28	0.4389	81	0.5624	134	0.1000	187	0.3833	240	0.6563
29	0.4035	82	0.4774	135	0.1000	188	0.4874	241	0.7119
30	0.3165	83	0.3194	136	0.4760	189	0.6171	242	0.7260

31	0.3143	84	0.2221	137	0.1769	190	0.1417	243	0.2489
32	0.1634	85	0.5185	138	0.4550	191	0.3886	244	0.3217
33	0.6801	86	0.4302	139	0.6524	192	0.4752	245	0.3492
34	0.6377	87	0.5117	140	0.6003	193	0.5382	246	0.3762
35	0.5576	88	0.6275	141	0.4181	194	0.1778	247	0.4064
36	0.4846	89	0.4933	142	0.5706	195	0.3660	248	0.4039
37	0.4405	90	0.3631	143	0.4094	196	0.4807	249	0.4287
38	0.4024	91	0.5619	144	0.5360	197	0.5096	250	0.4605
39	0.3518	92	0.5030	145	0.4209	198	0.6462	251	0.4812
40	0.1896	93	0.3794	146	0.5203	199	0.7554	252	0.4694
41	0.6736	94	0.2893	147	0.5821	200	0.3849	253	0.4741
42	0.5888	95	0.5355	148	0.3234	201	0.4900	254	0.4967
43	0.5114	96	0.4282	149	0.4978	202	0.5239	255	0.5038
44	0.4729	97	0.5786	150	0.1867	203	0.6472	256	0.4864
45	0.4135	98	0.5486	151	0.4842	204	0.2501	257	0.4972
46	0.3100	99	0.4745	152	0.5233	205	0.3950	258	0.5139
47	0.7329	100	0.3282	153	0.5963	206	0.4807	259	0.4769
48	0.6676	101	0.5407	154	0.3951	207	0.5166	260	0.4839
49	0.5908	102	0.4369	155	0.5394	208	0.6547	261	0.6238
50	0.4947	103	0.5191	156	0.3282	209	0.7577		
51	0.4480	104	0.6391	157	0.5078	210	0.8070		
52	0.3260	105	0.4392	158	0.5948	211	0.4305		
53	0.7483	106	0.3433	159	0.1000	212	0.4857		

References

[1] Morgan, M. A.; Fisher, D. L.; Milne, E. A. Electromagnetic Scattering by Stratified Inhomogeneous Anisotropic Media. *IEEE Trans. Antennas. Propag.,* vol. 35, no. 2, 1987. https://doi.org/10.1109/TAP.1987.1144069.

[2] Cohen, D.; Shavit, R. Bi-Anisotropic Metamaterials Effective Constitutive Parameters Extraction Using Oblique Incidence S-Parameters Method. *IEEE Trans. Antennas. Propag.,* vol. 63, no. 5, p. 2071–2078, 2015. https://doi.org/10.1109/TAP.2015.2405078.

[3] Chern, R.-L.; Kuo, C.-Y.; Chang, P.-H. Wave Propagation in Bianisotropic Metamaterials: Angular Selective Transmission. *Opt. Express*, vol. 22, no. 21, p. 25710–25721, 2014. https://doi.org/10.1364/OE.22.025710.

[4] Christopher, M. L.; Samarth, B.; Owen, D. M.; Eil, Y. Adjoint Shape Optimization Applied to Electromagnetic Design. Opt. Express, vol. 21, no. 18, p. 21693–21701, 2013. https://doi.org/10.1364/OE.21.021693.
